# Prevalence and determinants of selected cardio-metabolic risk factors among people living with HIV/AIDS and receiving care in the South West Regional Hospitals of Cameroon: a cross-sectional study

**DOI:** 10.1186/s13104-018-3444-0

**Published:** 2018-05-16

**Authors:** Roland Cheofor Ngu, Simeon-Pierre Choukem, Christian Akem Dimala, Julius N. Ngu, Gottlieb Lobe Monekosso

**Affiliations:** 1 Health and Human Development (2HD) Research Network, Douala, Cameroon; 2 Department of Internal Medicine and Pediatrics, Faculty of Health Sciences, University of Buea, Buea, Cameroon; 3 Diabetes and Endocrine Unit, Department of Internal Medicine, Douala General Hospital, Douala, Cameroon; 4 Faculty of Epidemiology and Population Health, London School of Hygiene and Tropical Medicine, London, UK; 5 University of Alabama at Birmingham, Birmingham, USA; 6 Nuffield Department of Medicine, University of Oxford, Oxford, UK; 7 World Health Organization Headquarters, Geneva, Switzerland

**Keywords:** Diabetes, Hypertension, Obesity, HIV/AIDS, Cameroon

## Abstract

**Objective:**

Metabolic disorders and cardiovascular risk factors are not routinely assessed in the care of HIV patients in developing countries, known to have the highest disease burden. We described the prevalence and factors associated with major cardio-metabolic risk factors (obesity, diabetes and hypertension) in HIV/AIDS patients.

**Results:**

The prevalence of diabetes, hypertension and obesity were 11.3% (95% CI 8.10–15.43), 24.8% (95% CI 20.1–30.0) and 14.5% (95% CI 11.1–19.3) respectively. Central obesity and high alcohol intake were the factors significantly associated with diabetes mellitus, while central obesity and overweight/obesity were significantly associated with having hypertension. Short duration of antiretroviral therapy was the significant predisposing factor for obesity. On multivariate analyses, the only association observed was between central obesity and diabetes (Adjusted OR 2.52, 95% CI 1.01–6.30, P = 0.048). Conclusively, DM, HTN and obesity are highly prevalent in HIV/AIDS patients in the SWR hospitals of Cameroon, with that of DM and obesity being higher than that seen in the general population while that of HTN equaling that of the general population. Awareness of these data among clinicians involved in the management of these patients should be emphasized.

## Introduction

Although potent antiretroviral therapy has reduced human immunodeficiency virus/acquired immunodeficiency syndrome (HIV/AIDS)-related morbidity and mortality, concerns about treatment-related metabolic complications and cardiovascular diseases (CVD) have emerged [1]. These complications resemble metabolic and body composition abnormalities of the metabolic syndrome (MS) described with increasing frequency in the general adult population [2]. MS is a constellation of metabolic and physical abnormalities frequently associated with increased risk of insulin resistance and cardiovascular morbidity and mortality in the general population [2]. According to the new criteria of the international diabetic federation (IDF), MS is defined as the presence of central obesity plus any two of the four factors; raised triglycerides (TG) level, reduced high density lipoprotein cholesterol (HDL-c), raised blood pressure (BP) and raised fasting plasma glucose [3].

Studies conducted in developed nations so far, have provided information suggesting that certain combination antiretroviral therapy regimens, especially those that include protease inhibitors, are associated with elevated serum triglycerides, total cholesterol (TC), and low-density lipoprotein cholesterol (LDL-c) as well as insulin resistance. On the other hand, there has been little, or no effect observed on high density lipoprotein cholesterol. This dyslipidemia and insulin resistance are known to favour the development of atherosclerosis, thus potentially explaining the connection between highly active antiretroviral therapy (HAART) and adverse cardio-metabolic outcomes [4].

In sub-Saharan Africa (SSA), first-generation NRTIs are still widely recommended as first-line therapies in treatment protocols which report have shown to be associated with various adverse metabolic and cardiovascular effects [5, 6]. Furthermore, with the initiatives by governments to scale-up care for HIV/AIDS, several SSA countries, including Cameroon, now provide antiretroviral drugs free of charge [7]. Consequently, patients are increasingly being prescribed lifelong HAART and are therefore at risk of developing related metabolic disorders and premature CVD. Despite this, there is paucity of data in Africa, and Cameroon particularly, on the prevalence of these cardio-metabolic risk factors among HIV-infected patients. Thus, aim of this study, was to describe the prevalence and identify the risk factors of diabetes, hypertension and obesity among HIV/AIDS patients in the South West Regional Hospitals of Cameroon.

## Main text

### Methods

#### Study design, setting and population

The participants in this cross-sectional hospital-based study were patients living with HIV/AIDS and attending the treatment centers of the Buea and Limbe Regional hospitals in the South West Region of Cameroon. Activities in these centers include: HIV counseling and testing, education on lifestyle modification and diet, viral load and CD4 count tests to monitor the progress of the treatment, dispensation of antiretroviral drugs, education to ensure compliance to treatment and management of defaulters, and follow-up of opportunistic infections.

Study participants were recruited consecutively during their routine follow-up visits. They had to be aged 21 years or older and to sign an informed consent form to take part in the study.

### Sample size

The sample size for this study was calculated using the online software www.openepi.com developed by Emory University, USA [8]. Based on previous prevalence, we assumed the highest prevalence of these 3 conditions will be 20% [9–11]. Our minimum sample size was 246 but we recruited 311 patients to improve the power of our results.

### Data collection and definition of variables

Data on medical history, family history and risk factors of hypertension and diabetes were collected from recruited participants as self-reports except for their last CD4 count which was obtained from their medical records. Anthropometric and vital parameters measured included: height, weight, BP, waist circumference and fasting blood glucose (FBG). These parameters were assessed using standardized methods. FBG was done using a glucometer (GlucoDr super sensor-AGM-2200 allmedicus, code 2) and diabetes was defined by a FBG ≥ 126 mg/dl (using the American Diabetes Association criteria) [12] and/or a previous diagnosis of diabetes. The BP on each arm for each patient was measured twice using an electronic sphygmomanometer (Brand: DBPOWER) and the average systolic and diastolic BP for both arms were calculated. Hypertension was defined by an average systolic BP ≥ 140 or an average diastolic BP ≥ 90 [13] and/or a previous diagnosis of hypertension. BMI was calculated as; weight (kg)/height^2^ (m^2^). Obesity was defined as a BMI ≥ 30 kg/m^2^ and overweight by a BMI between 25 and 29.9 kg/m^2^ [14]. Excess alcohol intake was defined as alcohol consumption ≥ 21 units/week for men and ≥ 14 units/week for women [15], adequate exercise was defined as ≥ 30 min of moderate aerobic exercise for at least 3 times a week [16], central obesity was defined as a waist circumference ≥ 94 cm for males and ≥ 80 cm for females according to the IDF [3], short duration of HAART was taken as antiretroviral therapy use ≤ 60 months and low CD4 count defined as CD4 count ≤ 350 cells/mm^3^.

### Statistical analysis

Data were analyzed using Epi info™ version 7.1.1.14. Frequencies and means of socio-demographic and clinical characteristic of our participants were first obtained. Furthermore, we assessed associations between patient characteristics and, each of hypertension, obesity and diabetes using the Pearson’s Chi squared tests or their non-parametric equivalents where appropriate. Statistical significance was set at a P value < 0.05. After that, to adjust for confounders, a multivariable logistic regression models were built for hypertension, diabetes and obesity incorporating all factors with a P value < 0.25 from the Chi squared test [17].

## Results

### Demographic and clinical characteristics of the study participants

Of the 311 participants recruited, 83.9% (261) were females. The participants’ clinical and demographic characteristics are presented on Table 1. Ages ranged from 22 to 73 years with a mean age of 43.4 ± 10.6 years. Three hundred and two participants (97%) were on HAART and the most frequently used regimen was Zidovudine–Lamivudine–Nevirapine (62.3%) (Table 1**).**

**Table 1:** Socio-demographic characteristics of the study population (n = 311)

Characteristic	Total (n = 311)	Females (n = 261)	Males (n = 50)	P*
Age in years, mean (± SD)	43.4 (± 10.6)	41.7 (± 10.6)	45.9 (± 10.4)	0.010
Age, years				0.220
≤ 45	197 (63.3%)	169 (85.8%)	28 (14.2%)	
> 45	114 (36.7%)	92 (80.7%)	22 (19.3%)	
BMI, mean (kg/m^2^)	25.0 (± 4.7)	25.2 (± 4.8)	23.8 (± 3.7)	0.047
BMI categories (kg/m^2^)				0.580^a^
< 25	182 (58.5%)	158 (82.4%)	32 (17.6%)	
≥ 25 to < 30	84 (27%)	70 (83.3%)	14 (16.7%)	
≥ 30	45 (14.5%)	41 (91.1%)	4 (8.9%)	
HAART combinations (n = 302)				0.128
AZT + 3TC + EFV	10 (3.3%)	7 (70%)	3 (30%)	
AZT + 3TC + NVP	189 (62.6%)	164 (86.8%)	25 (13.2%)	
AZT + 3TC + NVP + TFV	1 (0.3%)	1 (100%)	0 (0%)	
TFV + 3TC + EFV	71 (23.5%)	54 (76.1%)	17 (23.9%)	
TFV + 3TC + LPV + IDV	1 (0.3%)	1 (100%)	0 (0%)	
TFV + 3TC + NVP	28 (9.3%)	25 (89.3%)	3 (10.7%)	
D4T + 3TC + NVP	2 (0.7%)	1 (50%)	1 (50%)	
CD4 count, mean (cell/mm^3^)	470.5 (± 260)	484.4 (± 266.8)	398 (± 208.5)	0.032
CD4 count, cell/mm^3^				0.060
CD4 count ≤ 350	95 (30.5%)	74 (77.9%)	21 (22.1%)	
CD4 count > 350	216 (69.5%)	187 (86.6%)	29 (13.4%)	

*AZT* zidovudine, *3TC* lamivudine, *EFV* efavirenze, *TDF* tenofovir, *IDV* indinavir, *D4T* stavudine, *LPV* lopinavir, *BMI* body mass index, *SD* standard deviation

* P value for comparison between males and females; data are presented as mean ± SD or counts (%)

^a^P value after fisher’s exact test

### Prevalence and factors associated with diabetes

Among the 311 participants, 35 had diabetes corresponding to a prevalence of 11.3% (95% CI 8.1–15.4). Central obesity and a high alcohol intake were found to be significantly associated with diabetes on bivariate analysis (P = 0.03 and P = 0.03 respectively). On multivariate analyses, only central obesity remained weakly significantly associated with diabetes in these participants (Adjusted OR: 2.52, 95% CI 1.01–6.30, P = 0.048) (Table 2).

**Table 2:** Risk factors for diabetes in 311 HIV/AIDS patients

Risk factors for diabetes mellitus	Participants (n = 311)	Diabetes	Unadjusted OR (95% CI)	P value	Adjusted odds ratio (95% CI)	P value*
Age in years			0.98 (0.47–2.02)	0.95	–	
> 45	114	13 (11.4%)				
≤ 45	197	22 (11.2%)	1 (Ref)			
Gender			1.36 (0.56–3.30)	0.50	–	
Male	50	7 (14%)				
Female	261	28 (10.7%)	1 (Ref)			
Body mass index			0.64 (0.31–1.29)	0.21	0.95 (0.41–2.21)	0.898
Overweight/obesity	129	18 (14%)				
Normal/underweight	182	17 (9.3%)	1 (Ref)		1(Ref)	
Central obesity			0.44 (0.21–0.96)	0.03	2.52 (1.01–6.30)	0.048
Yes	170	25 (14.7%)				
No	141	10 (7.1%)	1 (Ref)		1 (Ref)	
Exercise			1.90 (0.51–7.02)	0.33		
Adequate	16	3 (18.8%)				
Inadequate	295	32 (10.9%)	1 (Ref)			
Alcohol intake			4.22 (1.01–17.69)	0.03	0.27 (0.06–1.21)	0.086
Alcoholics	9	3 (33.3%)				
Non alcoholics	302	32 (10.6%)	1 (Ref)		1 (Ref)	
Smoking				0.38		
Yes	4	1 (25%)	0.37 (0.04–3.69)			
No	307	34 (11.1%)	1 (Ref)			
ART			1.02 (0.12–8.37)	0.99	–	
Yes	302	34 (11.3%)				
No	9	1 (11.1%)	1 (Ref)			
ART duration (months)			1.02 (0.47–2.21)	0.97	–	
Long duration	88	10 (11.4%)	1 (Ref)			
Short duration/Nil	223	25 (11.2%)				
CD4 count (cells/mm^3^)				0.61	–	
≤ 350	95	12 (12.6%)	0.82 (0.39–1.73)			
> 350	216	23 (10.7%)	1 (Ref)			
Family history			1.21 (0.52–2.82)	0.65	–	
Yes	62	8 (12.9%)				
No	248	27 (10.9%)	1 (Ref)			

*Ref* Reference group

* P value = the P value after adjusting for the other factors in Multivariate logistic regression analysis

### Prevalence and factors associated with hypertension

The prevalence of hypertension in our study population was 24.8% (95% CI 20.1–30.0). Patients with central obesity and overweight/obesity were more likely to have hypertension (P = 0.004 and P = 0.003 respectively). On multivariate analyses using logistic regressions, no factor was found to be significantly associated with hypertension (Table 3).

**Table 3:** Risk factors for hypertension in 311 HIV/AIDS patients

Risk factors for diabetes mellitus	Participants (n = 311)	HTN	Unadjusted OR (95% CI)	P value	Adjusted odds ratio (95% CI)	P*
Age in years			0.66 (0.39–1.11)	0.12	1.56 (0.89–2.74)	0.119
> 45	114	34 (29.8%)				
≤ 45	197	43 (21.8%)	1 (Ref)		1 (Ref)	
Gender			0.95 (0.47–1.93)	0.89	–	
Male	50	12 (24%)				
Female	261	65 (24.9%)				
Body mass index			0.46 (0.27–0.78)	0.003	1.76 (0.92–3.40)	
Overweight	129	43 (33.3%)				0.089
Normal/underweight	182	34 (18.7%)	1 (Ref)		1 (Ref)	
Central obesity			0.45 (0.26–0.78)	0.004	1.62 (0.82–3.18)	0.163
Yes	170	53 (31.2%)			1 (Ref)	
No	141	24 (17.0%)	1 (Ref)			
Exercise			0.69 (0.19–2.49)	0.57	–	
Adequate	16	3 (18.8%)				
Inadequate	295	74 (25.1%)	1 (Ref)			
Alcohol intake			0.87 (0.18–4.25)	0.86	–	
Alcoholic	9	2 (22.2%)				
Non Alcoholics	302	75 (24.8%)	1 (Ref)			
Smoking			3.09 (0.43–22.34)	0.24	–	
Yes	4	2 (50%)				
No	307	75 (24.4%)				
ART			2.69 (0.33–21.86)	0.34		
Yes	302	76 (25.2%)				
No	9	1 (11.1%)	1 (Ref)			
ART duration (months)			1.41 (0.81–2.46)	0.22	–	
Long duration	88	26 (29.6%)				
Short duration/Nil	223	51 (22.9%)	1 (Ref)			
CD4 count (cells/mm^3^)			0.89 (0.51–1.54)	0.67	–	
≤ 350	95	25 (26.3%)				
> 350	216	52 (24.1%)	1 (Ref)			
Family history			1.48 (0.88–2.49)	0.14	0.64 (0.37–1.10)	0.109
Yes	127	37 (29.1%)				
No	184	40 (21.7%)	1 (Ref)		1 (Ref)	

*HTN* hypertension

* P value = the P value after adjusting for the other factors in Multivariate logistic regression analysis

### Prevalence and factors associated with obesity

The prevalence of obesity in our study population was 14.5% (95% CI 10.9–19.0), and that overweight was almost twice this prevalence (27%) (Table 1). The factor associated with obesity in bivariate analyses was shorter duration of ART (P = 0.040). On logistic regression, there was weak evidence of an association between long ART duration and obesity (adjusted OR: 2.40, 95% CI 1.00–5.75, P = 0.049) (Table 4).

**Table 4:** Risk factors for obesity in 311 HIV/AIDS patients

Risk factors for obesity	Total participants (n = 311)	Obese	Unadjusted OR (95% CI)	P value	Adjusted odds ratio (95% CI)	P value*
Age			0.66 (0.33–1.32)	0.24	0.81 (0.39–1.69)	0.573
> 45 years	114	13 (11.4%)				
≤ 45 years	197	32 (16.2%)	1 (Ref)		1 (Ref)	
Gender			2.14(0.73–6.28)	0.16	2.33(0.78–6.94)	0.130
Male	50	4 (8%)				
Female	261	41 (15.7%)	1 (Ref)		1 (Ref)	
Exercise			1.19 (0.26–5.44)	0.82	–	
Adequate	16	2 (12.5%)				
Inadequate	295	3 (14.6%)	1 (Ref)			
Alcohol intake			0.32 (0.08–1.34)	0.10	0.26 (0.06–1.15)	0.075
Alcoholics	9	3 (33.3%)				
Non alcoholics	302	42 (13.9%)	1 (Ref)		1 (Ref)	
ART duration (months)			2.38 (1.02–5.55)	0.04	2.40 (1.00–5.75)	0.049
Long duration (61–216).	88	7 (8%)				
Short duration/Nil (0–60).	223	38 (17.1%)	1 (Ref)		1 (Ref)	
CD4 count				0.54	–	
≤ 350 cells/mm^3^	95	12 (12.6%)	0.80 (0.39–1.63)			
> 350 cells/mm^3^	216	33 (15.3%)	1 (Ref)			
ART				0.50	–	
Yes	302	43 (14.2%)	1.72 (0.35–8.56)			
No	9	2 (22.2%)	1 (Ref)			

*ART* antiretroviral therapy

* P value = the P value after adjusting for the other factors in Multivariate logistic regression analysis

## Discussion

There is paucity of data on the potential association between HAART and cardio-metabolic risk factors in Africa. This study aimed to determine the prevalence and risk factors of hypertension, diabetes mellitus and obesity in HIV/AIDS patients in Cameroon and discuss them with reference to their equivalents in the general population. We found a significantly elevated prevalence of these CVD risk factors in our study population. The prevalence of diabetes, hypertension and obesity were as high as 11.3, 24.8 and 14.8% respectively in these major semi-urban settings of the South West Region of Cameroon.

The observed prevalence of diabetes in our study population is significantly higher than the 4.9% estimated national prevalence of diabetes mellitus in the Cameroonian general population in 2013 [18]. This could suggest that HIV/AIDS patients are more likely to develop this CVD risk factor compared to the general population, with possible explanations to this finding being the reported effects of the chronic HIV infection, prolonged antiretroviral therapy, or simply differences in the clinical and socio-demographic characteristics of the study populations. Chronic HIV infection has been reported to contribute to insulin resistance and diabetes by up-regulating inflammatory chemokines involved in insulin regulation [19–21]. Likewise, antiretroviral therapy has increasingly been suggested to contribute to hyperglycemia and diabetes mellitus [20]. Nevertheless, we found no association between HAART use, prolonged duration of therapy and diabetes. Insufficient data from the former study hinders the assessment of differences in socio-demographic and clinical characteristics between the study populations as potential reasons the observed difference in the prevalence of diabetes. Central obesity, which is a known risk factor for insulin resistance was the only factor found to be significantly associated with diabetes in our study population. Central obesity is particularly important in patients on antiretroviral therapy since several drug classes are known to induce a redistribution of body fats with predominant central and visceral deposition [22–25]. Despite the higher prevalence of diabetes among these patients compared to the general population, it is worth noting that a similar prevalence (11.5%) was reported in the Unites States of America, in patients of different origin from those of our study [26]. An even higher prevalence of diabetes among these patients was found in the study by Diouf et al. (14.5%). The participants in that study, however, had been on HAART for a much longer duration with predominantly protease inhibitor regimens [27].

We recorded a prevalence of hypertension in HIV/AIDS patients similar to that earlier reported in the general population in Cameroon (24.6%) in 2003 in the CamBod study [28]. The chronic inflammatory state following infection with HIV has been reported to result in vasculitis, aneurysms of large vessels, impaired renal flow, renal failure, all contributing to the development of hypertension [29]. This prevalence of hypertension is similar to that reported by Ekali et al. in Cameroon [30]. Similarities in study designs, settings and participants probably explain these similar findings. Malaza et al. [11] in rural South-Africa who enrolled younger participants compared to ours, reported a lower prevalence of hypertension (19.5%). On the other hand, Gazzaruso et al. who had all HIV/AIDS participants on treatment, estimated a much higher prevalence of hypertension among these HIV/AIDS participants at 34.2% [31]. As earlier reported, we also observed higher proportions of hypertension with increasing BMI. Despite the significant association noted between BMI, central obesity and hypertension at bivariate levels of analysis, these associations were lost after controlling for other confounders, confirming the multifactorial etiology of this important CVD risk factor.

Unlike, the prevalence of hypertension in our study which was similar to that of the general population, that of obesity, as defined by the BMI, was higher than that of the general population (9.6%) [32]. A similar prevalence of obesity (14%) was found by Amorosa et al. in a study in Philadelphia [33], however, lower values have been reported in settings similar to ours such as in the study by Muhammad et al. (6.5%) in Nigeria [34]. In addition to HAART, raised CD4 count levels have been reportedly associated with obesity in previous studies [27].

As our study has shown, the prevalence of HTN, obesity and DM is high amongst HIV/AIDS patients. This should inform the health policy, clinical practice and well as HIV research to target these diseases in HIV patients in Cameroon and internationally which could be in the form of passing a policy to be implemented in the health system by clinicians or other health care providers as well as routine screening and monitoring of these conditions in HIV patients. In addition, more robust evidence research such as systematic review should be carried out to ascertain the Global burden of these diseases in HIV patients.

## Conclusion

The prevalence of diabetes mellitus, hypertension and obesity in HIV patients in the South West Regional (SWR) Hospitals of Cameroon is high, with that of diabetes and obesity being higher than in the general population and that of hypertension equally that of the general population. Central obesity the only factor found to be marginally associated with diabetes. Awareness of these rising trends in these CVD risk factors in HIV/AIDS patients among clinicians is important in patient monitoring and management.

## Limitations

The interpretation of our results must, nevertheless, take into account some limitations among which the cross-sectional design of the study that hinders inferences on causality and the reliance on single BP and glucose measurements to ascertain disease status. Despite these limitations, this study remains an important contribution to the scanty literature on this topic by providing baseline epidemiological data for future research and health policy formulation.

## Abbreviations

AIDs: acquired immunodeficiency syndrome
BMI: body mass index
BP: blood pressure
CD4: cluster of differentiation
CVD: cardiovascular disease
DM: diabetes mellitus
FBG: fasting blood glucose
HAART: highly active antiretroviral therapy
HDL-c: high density lipoprotein cholesterol
HIV: human immunodeficiency virus
HTN: hypertension
IDF: international diabetic federation
LDL-c: low-density lipoprotein cholesterol
MS: metabolic syndrome
NRTIs: nucleotide reverse transcriptase inhibitors
OR: odds ratio
SSA: sub-Saharan Africa
SWR: South West Region
TC: total cholesterol
TG: triglycerides
